# Co-infection of Epstein-Barr virus and human papillomavirus in human tumorigenesis

**DOI:** 10.1186/s40880-016-0079-1

**Published:** 2016-01-22

**Authors:** Ying Shi, Song-Ling Peng, Li-Fang Yang, Xue Chen, Yong-Guang Tao, Ya Cao

**Affiliations:** Cancer Research Institute, Central South University, Changsha, 410078 Hunan P. R. China; Key Laboratory of Carcinogenesis and Cancer Invasion, Ministry of Education, Changsha, 410078 Hunan P. R. China; Key Laboratory of Carcinogenesis, National Health and Family Planning Commission, Changsha, 410078 Hunan P. R. China

**Keywords:** Epstein-Barr virus, Human papillomavirus, Co-infection, Nasopharyngeal carcinoma, Cervical cancer, Breast cancer, Prostate cancer

## Abstract

Viral infections contribute to approximately 12% of cancers worldwide, with the vast majority occurring in developing countries and areas. Two DNA viruses, Epstein-Barr virus (EBV) and human papillomavirus (HPV), are associated with 38% of all virus-associated cancers. The probability of one patient infected with these two distinct types of viruses is increasing. Here, we summarize the co-infection of EBV and HPV in human malignancies and address the possible mechanisms for the co-infection of EBV and HPV during tumorigenesis.

## Introduction

Oncogenic viruses include various classes of DNA and RNA viruses that are necessary but not sufficient for the development of all types of malignancies. A unifying theme is that cancer develops in a minority of persistently infected people and only after many years of chronic infection [1]. The majority of these cancers result from infection with an oncovirus. Viral infections contribute to approximately 12% of cancers worldwide, the vast majority (>85%) of which occurs in developing countries and areas [2, 3]. Epstein-Barr virus (EBV) and human papillomavirus (HPV), which are DNA viruses, were reported to be linked with 38% of all virus-associated cancers [4]. EBV is an oncogenic gamma-1 herpesvirus that infects more than 90% of the adult population worldwide, and it has powerful transforming potential for B lymphocytes in vitro, contributing to several lymphoid malignancies, including B, T, and natural killer (NK) cell lymphomas and epithelial carcinomas such as nasopharyngeal carcinoma (NPC) [4–9]. The viruses associated with the greatest number of cancer cases are HPVs, which cause cervical cancer and several other epithelial malignancies [10].

The probability of a single patient who is infected with two or more distinct types of viruses is increasing [11, 12]. Viral infections within carcinoma cells are not mutually exclusive. In the last 20 years, substantial attention has been paid to the observation that human immunodeficiency virus (HIV)-infected patients have a high risk of developing some cancers such as cervical carcinoma and some non-Hodgkin lymphomas [12]. Notably, cancers arising in the context of HIV-induced immunosuppression are usually associated with subsequent infection by oncogenic viruses such as EBV and HPV [13]. However, the detailed mechanism of the cooperation remains largely unknown, and further studies are needed to address whether this cooperation plays a critical role in tumor development.

The implication that HPV has a role in the carcinogenesis and prognosis of cervical cancer is well established. However, many studies have found that HPV infection is linked to NPC as an etiologic factor [14–17] and that EBV is also present in cervical cancer [18, 19] and prostate cancer [20]. The co-existence of both EBV and HPV has also been reported in other epithelial cancers, such as breast cancer [21]. The co-infection of EBV and HPV plays an important role in the initiation of a neoplastic transformation of carcinogenesis [22]. Interestingly, there is an interaction between EBV and HPV (whole viruses) in vivo as well as an interaction between HPV and EBV oncoproteins [e.g., latent membrane protein 1 (LMP1) reduces apoptosis in vitro] [23]. Whether the host is co-infected with two viruses, such as EBV and HPV, is unknown. Here, we discuss the co-infection of EBV and HPV in human malignancies and possible common characterizations in the molecular mechanisms of tumorigenesis.

## Co-infection of EBV and HPV in NPC

NPC is a common tumor in South China and Southeast Asia, but it is a rare disease among Caucasians [24, 25]. For many decades, the major etiologic factors proposed for NPC pathogenesis have included genetic susceptibility, environmental factors, and EBV infection [26]. However, although EBV infection is regarded as the primary factor for NPC, NPC patients are occasionally EBV-negative [27, 28]. Over the last decade, several studies have reported that HPV also exists in some EBV-negative NPC patients [14–17, 29]. HPV is considered one of the co-factors because it can transform epithelial cells, and the HPV DNA level is high in NPC biopsies.

The co-infection of EBV and HPV is usually found in NPC patients from endemic regions. In one study, NPCs in Iranian patients were detected by in situ hybridization, and a low percentage (15%) of EBV-positive NPC patients had HPV sequences (HPV type 6/11 or HPV type 16/18) [30]. Approximately 34% of NPC patients from Morocco had co-infection of HPV and EBV [31]. The co-infection of EBV and HPV was found in 47.7% of 88 Chinese NPC patients, and the “high-risk” types, including HPV types 16 and 18, accounted for 66.7% of 45 HPV-positive samples [32]; EBV was associated with types 2 and 3 NPC [the World Health Organization (WHO) classification] [33], which are non-keratinizing-type NPC, indicating that EBV infection might facilitate HPV infection.

However, co-infection of EBV and HPV is less common in NPC patients from some regions in the United States compared with that from the South China. HPV might be the etiologic factor in some EBV-negative, non-keratinizing NPC patients, including Caucasian North American patients [17]. A recent study in a population with a low incidence of NPC reported that both HPV-positive and EBV-positive NPC patients had similar overall survival (OS), whereas the HPV-positive, EBV-negative NPC group had shorter OS [28], which supported the etiologic role of HPV in NPC. Dogan et al. [28] reported that HPV and EBV infections were mutually exclusive in a low NPC incidence population; however, HPV positivity was significantly associated with WHO grade 2 tumors [34]. Numerous clinical studies reported that HPV type 18 infection was detected in NPC tumor tissues (mainly WHO type II) [14, 16, 17, 30, 31, 35, 36]; moreover, HPV types 16, 31, 35, 45, and 48 were detected in Moroccan patients with NPC type III [31]. As a result, HPV infection may act alone and have an etiologic role in the development of non-endemic, EBV-negative NPC, which is consistent with the results of the study of Caucasian North American patients with NPC [27] and another study of a United Kingdom population [37]. Both studies also indicated that EBV-negative NPC were associated with worse outcomes independent of HPV infection compared with EBV-positive NPC. Additionally, EBV-negative NPC in Caucasian patients was associated with smoking and the presence of HPV [38]. In contrast, EBV-negative NPC was not associated with HPV infection in Chinese patients [38, 39].

Using established EBV-negative NPC cell lines and advanced molecular biology techniques, our understanding of molecular alternations in these cells has been tremendously improved [40–43]. Until recently, only a few NPC cell lines, such as C666-1, that stably harbor the EBV genome have been used as EBV-positive models [44]. Other cell lines, such as CNE2, 5-8F, and 6-10B, may be EBV-positive in early cell culture passages, but the EBV genome was lost after subsequent passages. In these cells, it was determined that a single HeLa-related somatic cell hybrid was misidentified as an EBV-negative NPC cell line because the short tandem repeat profiles in NPC cell lines were not completely matched with that in HeLa cells [45]. Moreover, RNA sequencing showed that for HPV type 18-positive cervical adenocarcinoma cells, HeLa cells were a likely source of contamination in NPC cells CNE1 and HONE1 [46]. A new breakpoint and integration pattern has been observed and is being used to differentiate cells. It is also possible that these NPC cell lines derived from patients might have naturally acquired HPV infection prior to the establishment [16]. In contrast, low p53 expression was reportedly in HeLa cells, whereas CNE1 and HNE2 NPC cells had a high level of p53 expression [42, 47, 48]. Interestingly, these cells were not NPC type II, in which HPV type 18 is always detected [31]. HPV integrates into the host genome of HeLa cells [49] and cervical cancer tissues [50]; because EBV and HPV infections are mutually exclusive, it is easy to understand how the EBV genome could be lost during passaging these cells while HPV type 18 could still be detected. Moreover, HPV type 18 infection was not detected in other EBV-positive cells, such as AGS-EBV [51] and Akata-EBV [52], indicating that these cell lines might be used as good cell models for EBV-related research.

Co-infection of EBV and HPV is frequently found in endemic NPC, but it is less common in Caucasians than in Asian populations; HPV is an important etiologic factor in EBV-positive NPC among Caucasians [15]. Furthermore, co-infection of HPV and EBV may also cooperatively affect neoplastic transformation [53]. However, although HPV plays an etiologic role in EBV-negative NPC in Caucasians, more evidence is needed to confirm the roles of HPV in EBV-positive NPC in non-endemic regions. Moreover, several questions require further elucidation. First, there is a high risk of co-infection of EBV and HPV in some regions, such as South China, but the ratio of co-infection of EBV and HPV in NPC patients remains unclear. Next, it is also unclear which virus, either EBV or HPV, contributes to the first infection in co-infected patients. It will be interesting to address the difference in pathological and epidemiological characteristics among these NPC patients. Finally, the possible association of the HPV subtype and etiology in NPC requires further investigation. Of note, although genetic susceptibility of NPC in Asian ethnic groups has been well addressed [54–56], the association between genetic susceptibility factors and co-infection of HPV and EBV remains obscure.

## Co-existence of EBV and HPV in cervical cancer

Cervical cancer is the third most common malignancy in women and the fourth most common cause of death from cancer [57]. It is well known that there is a strong association between high-risk HPVs and cervical cancer [58]. Because it takes a long time for patients from being infected by HPV to developing cervical cancer, HPV is found in nearly 100% of cervical carcinoma cases; however, most women infected do not develop the disease, suggesting that other etiologies may be involved in the carcinogenesis of cervical neoplasm.

Many studies have found that there is a co-infection of high-risk HPV and EBV in cervical tissues [39, 59, 60], whereas some early reports did not report this finding [61, 62]. It would be of clinical significance to confirm whether EBV plays a causal role in cervical cancer development or if it is merely a bystander in the process.

EBV can transform cells bearing the EBV/C3d (third component of complement, C3) receptor, making them receptive to other oncogenic stimuli [63, 64]. These receptors are widely expressed on ecto- and endo-cervical biopsies of the uterine cervix [65]. This indicates that EBV can be a “helper” in the development of cervical cancer. Unexpectedly, EBV can be sexually transmitted [66] and can replicate in cervical cells [67]. Similarly, chronic cervicitis may also facilitate the EBV infection [68]. Women with cervical intraepithelial neoplasia 1 (CIN1) or greater are almost four times likely to be EBV-positive than women without the disease [68]. EBV prevalence is associated with older age and a diagnosis of CIN1 or greater. In cervical carcinoma, the co-infection rate was the highest (67%) in squamous cell carcinoma, whereas it was the lowest (7%) in normal cervical tissue [59]. The remarkable difference in EBV infection noted between normal cervixes and squamous cell carcinoma suggests that the co-infection of the two viruses in the cervix may facilitate cervical cancer progression and act as a marker of poor prognosis in patients with cervical cancer.

HPV types 16 and 18 are the two most common high-risk HPV types and were involved in the development of cervical cancer [69, 70]. HPV type 58 is relatively prevalent in China and other Asian countries, and genome-wide analysis confirmed its expression in cervical lesions [71–74]. The infection of both high-risk HPV types (such as HPV type 58) and EBV display considerable geographic variation, and, prior to performing epidemiologic studies, it is necessary to determine which HPV type might co-infect with EBV. Moreover, the clinical relevance of genital infection caused by EBV may be due to the virus’s capability for cellular transformation, which possibly contributes, as a co-factor, to the development of malignancy.

## EBV and HPV in breast cancer

Breast cancer is the second leading cause of cancer-related death in women around the world [75]. The etiology of human breast cancer is significantly affected by multiple risk factors (age, hormones, alcohol use, diet, familial history, etc.). As viruses are etiologic agents of some human cancers, researchers have become interested in identifying viral agents for breast cancer.

Interest in EBV as a possible breast agent arose from the presence of EBV in breast tissue [76]. Some researchers have also proposed that HPV is a candidate virus in breast cancer after finding that oncogenic HPV could immortalize human mammary epithelial cells [77]. Interestingly, HPV-16 E6/E7, the genotype that is most frequently detected in cervical uterine cancer, was the only genotype identified in HPV-positive breast cancer and integrated into the host genome in all cases. No HPV DNA sequences were found in invasive breast carcinoma [78, 79]. However, high-risk HPV types 16, 18, and 33 have been identified in breast cancers from widely different populations [80]. Clearly, HPV and EBV prevalence in mammary epithelial cells from breast cancer patients is relatively low; therefore, determining the direct etiologic role of these viruses is excluded.

EBV is a major contributor to Hodgkin lymphoma, and significant associations exist between the incidence of Hodgkin lymphoma and breast cancer, suggesting that EBV may act as an etiologic factor for some breast cancers [81]. Some EBV-positive cancer cells have the same histological characteristics as Reed-Sternberg cells, suggesting that EBV-positive lymphocytes infiltrate breast tissues and transmit EBV to breast epithelial cells [21]. Women with HPV-positive breast cancer at the age of diagnosis are younger than those with HPV-negative breast cancer [21], supporting the hypothesis that women with HPV-positive breast cancer may have sexually transmitted HPV [82].

Co-infection of EBV and HPV appears to be present in a significantly higher proportion in breast cancers than in normal breast epithelial cells [21, 83]. The Glenn group reported that HPV and EBV co-exist in several human cancers, and the presence of these viruses in breast cancer is associated with young age at diagnosis and, possibly, an increased breast cancer grade [21].

As many studies provide insufficient evidence, testing in larger sample groups is needed to demonstrate that EBV and HPV are associated with breast cancer [84]. To clarify whether the co-infection of EBV and HPV promotes the development of breast cancer, further studies are needed.

## EBV and HPV in prostate cancer and other malignancies

Among men, prostate cancer is the most common cancer and the second leading cause of cancer-related death [85]. Because HPV is always detected in both benign and malignant prostate tissues, it is considered an important risk factor for prostate cancer [86].

Although EBV is a ubiquitous virus with known oncogenic potential, few investigations have demonstrated its association with prostate cancer. Several reports showed that EBV was present in prostate tissues. In Sweden, EBV was present in 8.8% (31 of 352) of benign and malignant prostate tissues [87], and in the United States, EBV was present in 8% (16 of 200) of all normal, benign, and malignant prostate tissues [88]. In another study, approximately 37% (7 of 19) of prostate cancer patients had EBV infection [89].

Both HPV type 18 and EBV gene (EBNA1) sequences were identified in a high and approximately equal proportion of normal, benign, and prostate cancer specimens [20]. The sequences were located in the nuclei of prostate epithelial cells. These observations are consistent with those in studies of high-risk HPVs [86] and studies of EBV in prostate cancer [20, 88, 89]. The frequency of co-infection of EBV and HPV was significantly higher in prostate cancer (55%) than in benign (15%) and normal prostate (30%) specimens. Additionally, experimental evidence shows that HPV and EBV work together to promote the proliferation of cultured cervical cells [23], suggesting the same may be true for prostate epithelial cells.

We summarized the ratio of co-infection of EBV and HPV in several malignancies (Table 1). It is possible that HPV exerts its oncogenic influences in concert with co-factors, including a possible collaboration with EBV. Although the prostate is a niche for multiple viral and other infections, some of which have oncogenic potential, few investigations have focused on the relationship between prostate cancer and viral infection. Increasing data are needed to clarify the role that the co-infection of HPV and EBV plays in the carcinogenesis of prostate cancer.

**Table 1:** Summary of co-infection of EBV and HPV in several malignancies

Tumor type	Co-infection	Subtype of HPV	EBV product(s)	Reference
NPC	15% (Iran)	6/11, 16/18	EBER	[30]
	34% (Morocco)	16, 31, 45, 48	EBER	[31]
	42% (China)	18	EBER	[32]
Cervical cancer	31.8%	16, 18, 58	EBER	[39]
	12.5%–69%	16, 18	BALF1, BARF-1, LMP1	[59]
	21.2%–64.3%	16, 18	EBNA3B	[60]
Breast cancer	35%	16, 18, 33	LMP1, EBNA-1	[21]
Prostate cancer	8.8% (Sweden)	18	EBER	[88]
	37% (United States)	18	EBER, EBNA-1	[89]

*EBV* Epstein-Barr virus; *HPV* human papillomavirus; *NPC* nasopharyngeal carcinoma; *EBER* EBV-encoded RNA; *BALF1* *Bam*H1 A fragment leftward reading frame 1; *BARF-1* *Bam*H1-A right frame 1; *LMP1* latent membrane protein 1; *EBNA3B* EBV nuclear antigen 3B; *EBNA-1* EBV nuclear antigen

Lung cancer is the leading cause of cancer-related death in the world, and it is unlikely that HPV or EBV are important etiologic agents in lung adenocarcinoma, even among people who have never smoked [90]; in fact, in one study, only 1 of 219 patients with lung squamous cell carcinoma tumors was positive for HPV 16 [91]. Gastric carcinoma is the second leading cause of cancer-related death in developed and developing countries, and it has a widely varying geographic incidence [92]. In one study, RNA sequencing demonstrated that only 5.6% of gastric carcinoma cases harbored EBV-encoded transcripts, and none of the tumors showed evidence of EBV integration into the host genome [91]. Another report indicated that EBV integration was found in approximately 9% of malignant epithelial cells in gastric carcinoma [93].

Esophageal squamous cell carcinoma (ESCC) is prevalent worldwide and is particularly common in certain regions in Asia, including Southeast China. One group of researchers showed that EBV infection contributed to the molecular pathogenesis of ESCC [94], whereas other groups indicated that EBV was not associated with ESCC [95, 96]. On the basis of the causal relationship between HPV infection and the carcinogenesis of ESCC, serological evidence from a high-incidence area of China further supports that ESCC is infected with HPV type 16 [97–99]; however, this finding contradicted that of another group [100]. Clearly, the prevalence of co-infection of EBV and HPV in ESCC requires further study.

Altogether, it is unclear whether the co-existence of EBV and HPV acts as a promoter or bystander in the development of these carcinomas aforementioned. To improve the treatment and prognosis, it is important, from the standpoint of genetic etiology, to clarify the connection.

## APOBEC3 deaminases may promote co-infection of EBV and HPV

Oncogenic viruses induce cancer by a variety of mechanisms that do not include the viral episome or lytic replication; for example, the immune system can have a deleterious or protective role, with increasing immunosuppression in human virus-associated cancers and with activating DNA damage response [4, 101]. Recently, a family of proteins that includes the apo lipoprotein B mRNA editing enzyme catalytic polypeptide 3 (APOBEC3) was well characterized due to the ability to provide innate immunity from a variety of exogenous pathogens, including viral infection [102, 103]. Cytidine deaminases of the APOBEC3 family all have specificity for single-stranded DNA, which may become exposed during replication or transcription of double-stranded DNA. Three human APOBEC3A (hA3A), hA3B, and hA3H genes are expressed in keratinocytes and the skin; all of them induce hypermutation in HPV16 DNA upon beta interferon stimulation in benign and precancerous lesions during natural viral infection [104–106]. Interestingly, APOBEC3 is capable of editing both EBV and HPV by inducing cytidine-to-uracil mutations in viral DNA [107]. In one study of early-stage breast carcinogenesis, HPV type 18 infection induced APOBEC3B activity, leading to genome instability [108], whereas in another study APOBEC3 induced E2 hypermutation in HPV16 DNA upon beta interferon stimulation in cervical keratinocytes [105]. In HPV-induced tumorigenesis, APOBEC3 also mutated other host genomic regions, including those of phosphatidylinositol-4,5-bisphosphate 3-kinase, catalytic subunit alpha (PIK3CA) helical domain [109]. Interestingly, EBV-positive gastric cancer displayed recurrent PIK3CA mutations [110]. It is possible that APOBEC3 increases the chance of viral DNA integration in the host by inducing mutations and genome instability after viral infection.

## Perspectives and conclusions

Studies on the roles of chromatin in the regulation of viral infections have revealed the significance of dynamic viral-host chromatin interactions in determining viral infections as well as the interplay between viral-encoded proteins in modulating chromatin. These interactions are especially significant for multiple epigenetic events, such as chromatin assembly, histone and DNA modifications, and higher-order chromosome structures that control viral episome generation and maintenance after viral infection [111–113]. Two scenarios of cooperation between EBV and HPV after co-infection in cancer development should be further addressed; the detailed epigenetic mechanism of this cooperation remains largely unknown, and further studies are necessary to elucidate whether co-infection of EBV and HPV plays any role in the development of malignant tumors. Moreover, chromatin control of viral co-infection also represents a new field with potential targets for the development of novel antiviral therapies. Of note, most arguments of co-infection are mainly supported only by polymerase chain reaction data and in situ hybridization; additional evidence of co-infection of both EBV and HPV or gene products to the same cells or tissues should be addressed.

The effect of such reprogramming on the viral life cycle also remains unknown; it will be interesting to evaluate the host-virus interactions through combined genome-wide analyses. Recently, a large-scale functional genomic analysis of EBV, together with over 700 publicly available high-throughput sequencing data sets for human lymphoblastoid cell lines, were mapped to the EBV genome, and viral lytic genes were found co-expressed with cellular cancer-associated pathways, indicating that the lytic cycle may play an unexpected role in virus-mediated oncogenesis [114, 115]. Moreover, a comprehensive resource describing a systematic quantitative analysis of temporal changes in the host and viral proteins throughout the course of a productive infection could provide dynamic views into virus-host interactions [116]. Further studies on genome-wide approaches will lead to more comprehensive models of viral persistence and its role in the interplay between co-infection of EBV and HPV and the infected cells.

Oncogenic virus-induced cancers are an important global concern. Remarkable progress continues to be made in the global fight against cancer by preventing the spread of these viruses, by developing and distributing safe and effective vaccines against them, and by identifying the oncogenic mechanisms used by these viruses, enabling the development of new treatment options for these virally associated human malignancies. Although there is a vaccine for HPV [117], there is not one for EBV (50 years after EBV was identified). Now, two preventive vaccines for HPV types 16/18 and HPV types 6/11/16/18 are commercially available in more than 140 countries and regions, and the implementation rate of these two HPV vaccines has reached approximately 80% among suitable women in Australia. Interestingly, DZ1 is regarded as a phosphorothioate-modified “10–23,” DNAzymes were specifically targeted to the LMP1 mRNA, and DZ1 treatment increases the radiosensitivity of NPC to standard radiotherapy [118–125]. The DZ1 treatment is safe and has been shown to be efficacious in different modes, indicating the potential for DZ1 therapeutic approaches for the treatment of EBV-related cancers.
